# Light-triggered chemical amplification to accelerate degradation and release from polymeric particles†
†Electronic supplementary information (ESI) available. See DOI: 10.1039/c5cc06143a



**DOI:** 10.1039/c5cc06143a

**Published:** 2015-10-08

**Authors:** Jason Olejniczak, Viet Anh Nguyen Huu, Jacques Lux, Madeleine Grossman, Sha He, Adah Almutairi

**Affiliations:** a Department of Chemistry and Biochemistry , University of California , San Diego , 9500 Gilman Dr. , La Jolla , California 92093 , USA; b Department of NanoEngineering , University of California , San Diego , 9500 Gilman Dr. , La Jolla , California 92093 , USA . Email: aalmutairi@ucsd.edu; c Skaggs School of Pharmacy and Pharmaceutical Science , University of California , San Diego , 9500 Gilman Dr. , La Jolla , California 92093 , USA; d Department of Materials Science and Engineering , KACST-UCSD Center of Excellence in Nanomedicine and Engineering , University of California , San Diego , 9500 Gilman Dr. , La Jolla , California 92093 , USA

## Abstract

We describe a means of chemical amplification to accelerate triggered degradation of a polymer and particles composed thereof. We designed a light-degradable copolymer containing carboxylic acids masked by photolabile groups and ketals. Photolysis allows the unmasked acidic groups in the polymer backbone to accelerate ketal hydrolysis even at neutral pH.

## Abstract 2

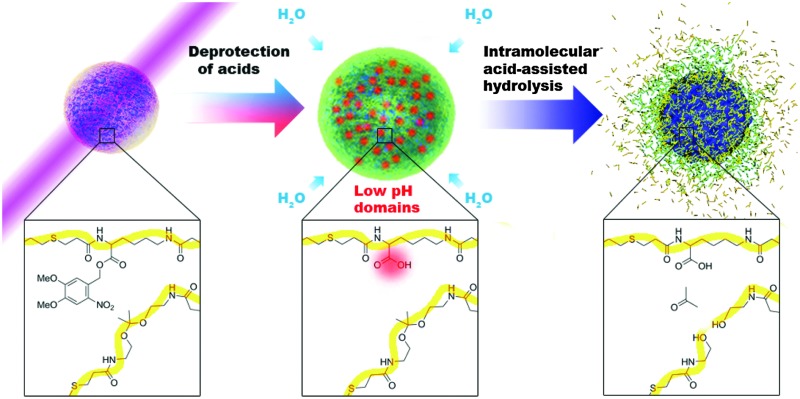
We describe a means of chemical amplification to accelerate triggered degradation of a polymer and particles composed thereof.

On-demand or environmentally triggered disassembly of polymers is a widely sought-after goal, as such materials would be tremendously useful in a broad range of industries, including healthcare, cosmetics, agriculture, and electronics.^1,2^ Despite this, few synthetic polymers degrade with high sensitivity in response to specific stimuli. Most current degradable materials are unresponsive to the often subtle changes found in biological systems or, in the case of photodegradable polymers, require long, intense irradiation that may not be biologically compatible. This limitation results from the fact that most of these materials convert one signalling event to only one chemical change, such as a single break in the polymer backbone^3–5^ or a change in hydrophobicity of one monomeric unit.^6,7^

Self-immolative polymers can amplify responses to stimuli *via* head-to-tail depolymerization and have thus been developed to circumvent this limitation.^8–10^ However, most of these materials rely on slow intramolecular rearrangements to degrade their backbone,^11–16^ ultimately slowing down depolymerization. Alternatively, self-immolative polymers containing more labile bonds have also been developed,^17,18^ but these bonds are likely not resilient enough to escape degradation in a physiological setting, even in the absence of the intended stimulus. The Phillips group has recently made substantial improvements to self-immolative polymers by creatively altering polymer backbones to maximize the effect of slow rearrangements^19,20^ and minimize nonspecific degradation,^21^ but there is still room to add to these strategies. Here, we have designed a polymer in which photocleavage unmasks acidic groups in the polymer backbone that then provide intramolecular assistance to ketal hydrolysis^22^ so that minimal signal, in this case brief, low-power UV irradiation, triggers significant polymer degradation. This strategy should allow faster release with less irradiation than existing light-degradable polymers.^23–25^

Our design was inspired by the extensive literature on rates and mechanisms of ketal hydrolysis,^21,22^ degradation rates of polyketals,^26^ and disassembly of ketal-modified polymeric particles.^27–31^ Ketal hydrolysis rates are known to vary with hydrophilicity,^32,33^ and water accessibility affects the kinetics of disassembly and degradation of polymeric particle assemblies containing ketals either within the backbone^34,35^ or as pendant groups.^36^ These findings inspired hydrophobic–hydrophilic switching mechanisms to exert further control over particle disassembly and/or degradation.^34^ More recently, our group observed rapid degradation of a polyketal due to intramolecular assistance of acids^21,22,36,37^ in a polymer designed as an MRI contrast agent.^37^ The degradation occurred much more rapidly (in hours) than in comparable hydrophilic polymers (in days)^26^ at the same buffered pH but containing no intramolecular acids. Here we employ the same concept to a light-degradable particle. We incorporate photoacids as pendant groups into a polyketal backbone (Scheme 1), from which we formulate particles. Cleavage of the photocage upon UV irradiation unmasks a carboxylic acid. This both releases acid groups in the vicinity of the backbone ketals (not necessarily adjacent along the backbone; polymer entanglement in a nanoparticle would juxtapose groups that would be distant from one another in dilute solution), and makes the polymer more hydrophilic, both of which facilitate ketal hydrolysis.

**Scheme 1:**
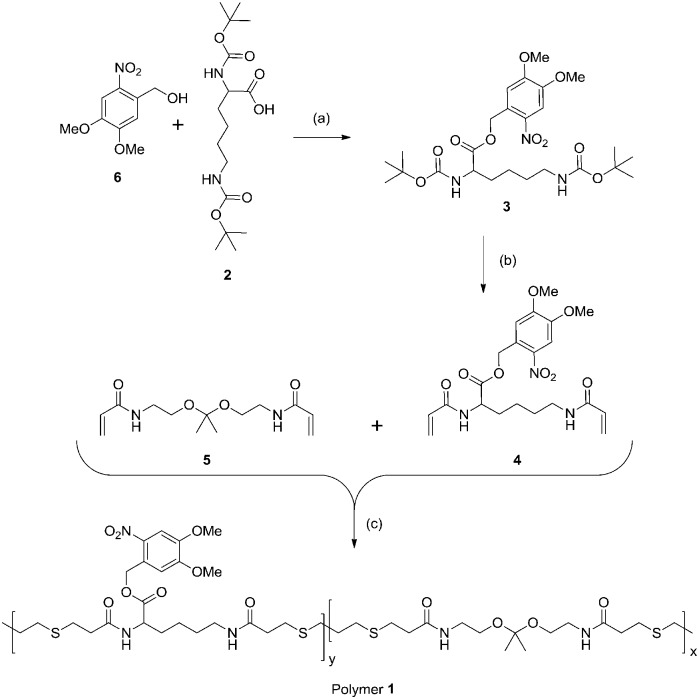
Synthesis of polymer **1**: (a) EDC, DMAP, DCM, (compound **2** used as the dicylohexylamine salt), 52%; (b) (i) TFA, DCM (ii) acryloyl chloride, Et_3_N, DCM, 0 °C, 49%; (c) **5**, 1,3-propanedithiol, Et_3_N, DMSO, 42%.

To synthesize a polymer containing both ketal moieties and protected acid functions, we prepared two monomers for copolymerization (Scheme 1). Ketal monomer **5** was prepared by established methods.^26^ To synthesize the monomer bearing a protected acid, **2** was esterified with alcohol **6** to form **3**. *ortho*-Nitrobenzyl alcohol **6** was chosen as a photolabile protecting group due to its commercial availability, relatively high tolerance to subsequent reactions, and its well-characterized photochemistry.^38,39^ Though **6** has limitations as a photolabile group (low tissue penetration of UV light for drug delivery applications) it and related protecting groups have been used for cell studies^40–42^ and creative drug delivery methods in mammals.^43,44^ Deprotection of the amines of **3** and treatment with acryloyl chloride gave **4**. Monomers **4** and **5** in equal proportions were copolymerized using a Michael addition with 1,3-propanedithiol to yield polymer **1** with weight average molecular weight (*M*_w_) 13 900 Da and a polydispersity index (PDI) of 1.71 by gel permeation chromatography (GPC) relative to poly(methyl methacrylate) standards (Fig. S1, ESI†). The monomers were incorporated equally as seen by ^1^H NMR spectroscopy (Fig. S2, ESI†). Though it leads to relatively high PDI Michael addition proved to be an ideal means of polymerization due to its relatively mild conditions, a necessity to avoid degradation of the ketal.

Polymer degradation was monitored using ^1^H NMR spectroscopy by following hydrolysis of the ketal to determine the degradation rate (Fig. 1a and b). Polymer **1** was dissolved in a 9 : 1 mixture of deuterated DMSO and deuterated phosphate buffer at pH 7.4 and phosphate solution at pH 5 and irradiated for times ranging from 0 to 20 minutes with UV light (1.35 mW cm^–2^). Irradiation and release of acids did not noticeably change the pH of either solution. Though the high proportion of organic solvent slows ketal hydrolysis by orders of magnitude,^45,46^ DMSO was required to solubilize the polymer prior to irradiation. Following irradiation substantial amounts of the light-sensitive protecting groups still appeared intact; by ^1^H NMR only 50% of the acids were exposed even after 20 min of irradiation (Fig. S3A, ESI†). The samples were then monitored by ^1^H NMR spectroscopy at various time points throughout incubation at 37 °C. Although the ketal peak diminished and the acetone peak grew (Fig. 1c), the percentage of hydrolyzed ketal over time could not be accurately determined because of signal overlap. Ketal hydrolysis was instead followed by conversion of the methylene protons (Fig. 1a, protons A) vicinal to the ketal into protons vicinal to an alcohol. The initial rate of ketal hydrolysis was determined for each condition (Fig. 1b).

**Fig. 1:**
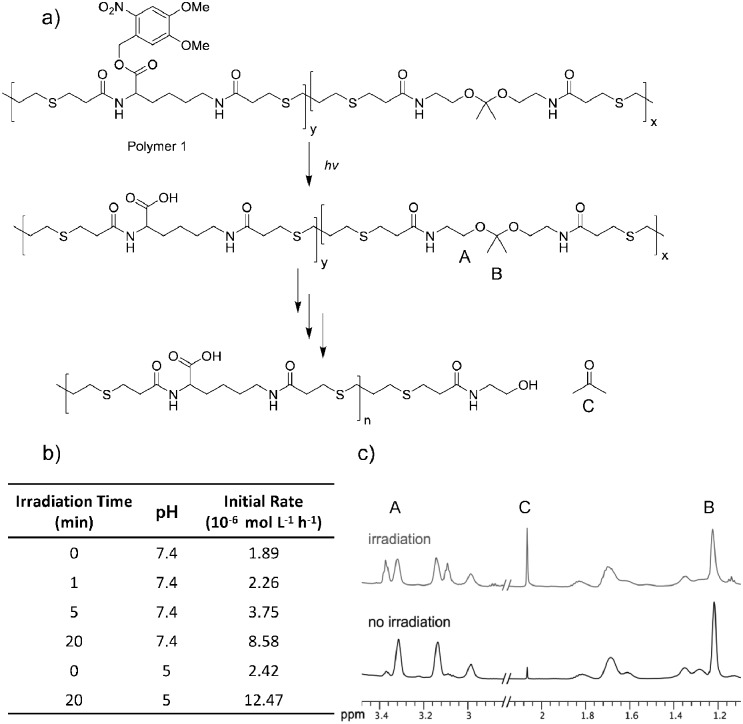
(a) Degradation scheme of polymer **1**. (b) Initial rate of ketal hydrolysis at varying pH and with varying amounts of irradiation. (c) ^1^H NMR spectra of polymer samples after 23 days at pH 7.4 with 20 min UV irradiation (top teal) or without irradiation (bottom black). Rates and ^1^H NMR spectra were obtained in a 9 : 1 mixture of DMSO to aqueous solution.

The initial rate of hydrolysis at pH 7.4 increased with longer irradiation times, becoming four times faster after 20 minutes of irradiation than with no irradiation (Fig. 1b). Irradiation for only 5 min caused the pH 7.4 degradation kinetics to be 55% faster than the pH 5.0 degradation kinetics without irradiation. Comparable polymers containing the same ketal moiety have a half-life of roughly 1 h at pH 5 in solutions with a smaller proportion of organic solvents, suggesting that this polymer would degrade even more rapidly in biological settings.^26^ A control polymer with benzyl protecting groups (removable by hydrogenation), polymer **9**, was synthesized (Fig. S9, ESI†) to ensure that degradation was accelerated by release of acids. No substantial difference in rate was observed between irradiated and untreated polymer **9**. In contrast, degradation was accelerated when roughly 50% of the acids of polymer **9** were exposed by hydrogenation (Fig. S11, ESI†).

Polymer degradation was also assessed by GPC (Fig. S3, ESI†). The immediate shift to longer retention times observed upon irradiation of samples of polymer **1** is too rapid to indicate degradation. Instead, it likely results from a change in hydrophilicity caused by release of acids, increasing interactions with the column material. Shifts towards longer retention times in subsequent time points do support polymer degradation in support of the NMR spectroscopy experiments.

To examine whether this degradation strategy allows rapid light-triggered release, we formulated nanoparticles of polymer **1** by single emulsion encapsulating the model payloads fluorescein diacetate (FDA) or Nile red (size = 193 ± 23 nm). We first examined light-triggered release by measuring fluorescence quenching of encapsulated Nile red. Nile red is fluorescent in the hydrophobic environment of nanoparticles, but its fluorescence is quenched in aqueous environments. Rapid fluorescence quenching was observed upon irradiation of particles suspended in pH 8.0 tris buffer (Fig. 2a). This quenching indicates substantial changes in morphology, allowing Nile red escape or entry of water into the particles. Particle degradation was assessed following irradiation and subsequent incubation at 37 °C by dynamic light scattering (DLS) with fixed attenuation. Upon UV irradiation, count rate decreased substantially and the PDI increased within 4 h, indicating substantial changes in particle morphology and possible degradation (Fig. 2b). Particles remained relatively stable in the absence of irradiation. The morphological changes were further examined by transmission electron microscopy (TEM) (Fig. 2c). After irradiation, subsequent incubation for 4 h, and drying particles appeared to disintegrate (Fig. 2d).

**Fig. 2:**
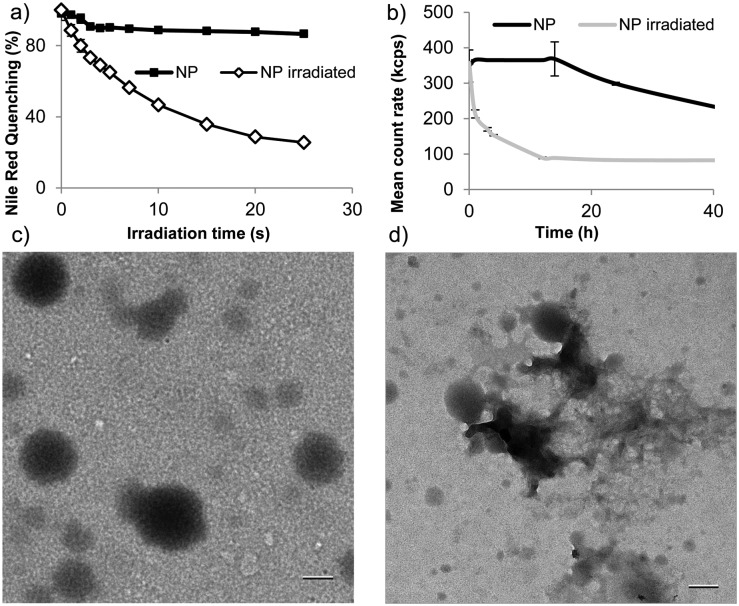
(a) Quenching of fluorescence of Nile red encapsulated in nanoparticles of polymer **1** following irradiation with UV light. (b) Count rate of nanoparticles after irradiation 5 min (35 mW cm^–2^, *λ* = 320–480 nm) by DLS. (c) Representative TEM micrographs of particles prior to irradiation and (d) Post-irradiation 5 min (35 mW cm^–2^, *λ* = 320–480 nm) and incubation at 37 °C for 4 h (scale bars = 200 nm).

To confirm payload release from nanoparticles, Raw 264.7 mouse macrophage cells were incubated with particles containing FDA (Fig. 3a) and irradiated for 5 min with UV light (10 mW cm^–2^) (Fig. 3b). This is a comparable power and shorter irradiation time than has been used with materials incorporating this photocage in cellular studies.^47,48^ FDA is a non-fluorescent molecule hydrolyzed by intracellular esterases to form fluorescent fluorescein; only released FDA would encounter these esterases. UV irradiation led to high intensity fluorescence, while non-irradiated cells did not fluoresce appreciably (Fig. 3c). This demonstrates that nanoparticles composed of polymer **1** release cargo in the presence of cells under irradiation conditions that have minimal impact on cellular viability (the viability of cells irradiated with particles is confirmed by MTT assay (Fig. S8, ESI†)).

**Fig. 3:**
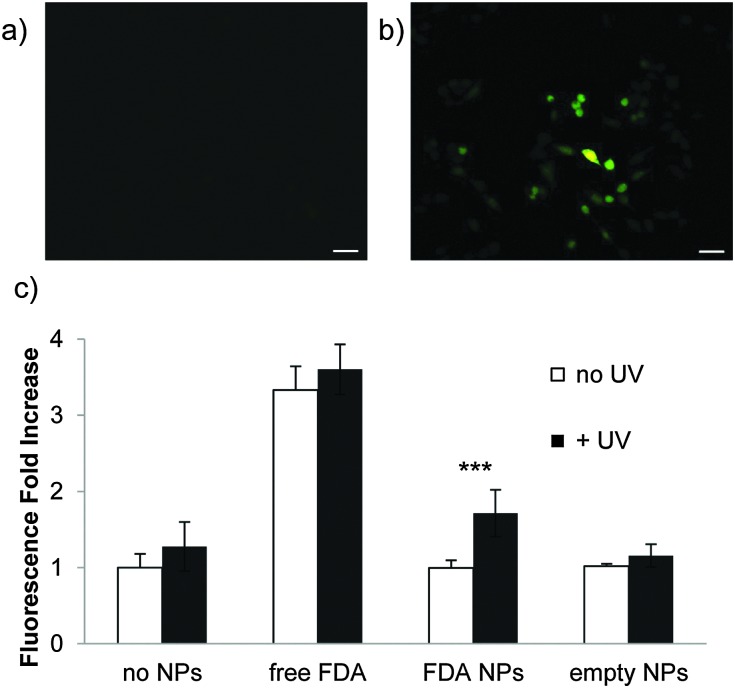
(a) Raw 264.7 mouse macrophage cells incubated (30 min, 37 °C) with nanoparticles (a) in the absence of irradiation and (b) irradiated for 5 min (10 mW cm^–2^). Scale bars = 30 μm. (c) Increase in FDA fluorescence; *p* < 0.001.

Finally, we assessed cellular compatibility by MTT assay in Raw 264.7 mouse macrophage cells after treatment with empty nanoparticles irradiated prior to treatment (Fig. 4), irradiated after incubation with cells (Fig. S8, ESI†), not irradiated, and polymer **1** (Fig. S7, ESI†). Neither nanoparticles nor polymer significantly impacted mitochondrial activity up to 200 μg mL^–1^, suggesting polymer **1**'s potential for drug delivery. Particle degradation products also had less effect on cellular viability than intact nanoparticles (Fig. 4).

**Fig. 4:**
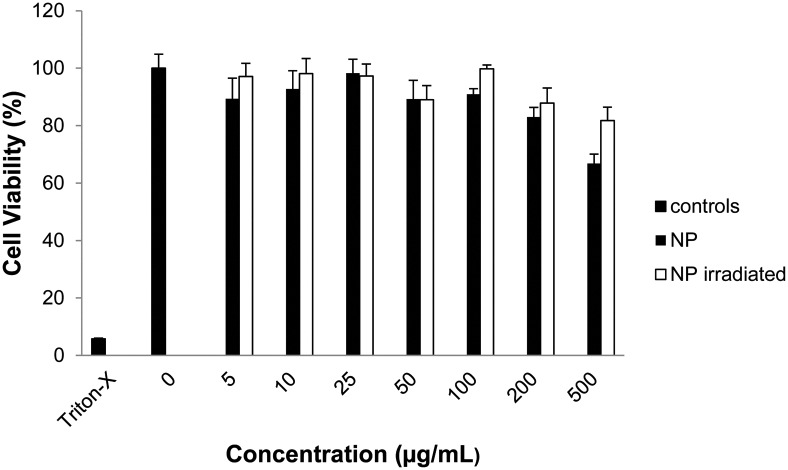
Nanoparticles of polymer **1** are well-tolerated by Raw 264.7 macrophages. MTT assay following 24 h incubation with nanoparticles, either intact or pre-irradiated for 5 min with UV light (10 mW cm^–2^).

Herein we have demonstrated that unmasking acids in a polymer backbone to accelerate the hydrolysis of ketals at neutral pH is a viable strategy to accelerate polymer and particle degradation. Rapid light-triggered release from polymer **1** nanoparticles demonstrates the potential of this strategy for triggered degradation in general; other chemical groups could be employed to confer responsiveness to other stimuli.

The authors gratefully acknowledge the NIH New Innovator Award (DP 2OD006499) and KACST-UCSD Center for Excellence in Nanomedicine and Engineering for funding. NMR spectra were acquired at the UCSD Skaggs School of Pharmacy and Pharmaceutical Sciences NMR facility. The authors would also like to thank Jessica Moore, Minnie Chan, Amy Moore, Carl-Johan Carling and Brendan Duggan.
