# Supplementary material for: Light-triggered chemical amplification to accelerate degradation and release from polymeric particles
Source: Chem Commun (Camb). 2015 Oct 8;51(95):16980–3. doi: 10.1039/c5cc06143a (PMC4819761; doi:10.1039/c5cc06143a)
Supplement: Supplementary file 1 [file CC-051-C5CC06143A-s001.pdf]

Electronic Supplementary Material (ESI) for ChemComm.
This journal is © The Royal Society of Chemistry 2015

Supporting Information

Light-Triggered Chemical Amplification to Accelerate Degradation and Release from Polymeric Particles

Jason Olejniczak, Viet Anh Nguyen Huu, Jacques Lux, Madeleine Grossman, Sha He, Adah Almutairi

Table of contents:

1. General methods and instrumentation
2. Abbreviations
3. Synthesis of polymer **1**
4. Degradation of polymer **1**
5. Particle formulation and characterization
6. *In vitro* release from particles
7. Cytotoxicity assays
8. Synthesis of control polymer **9**
9. Degradation of control polymer **9**
10. NMR spectra
11. Additional TEM images
12. SEM images

1. General methods and instrumentation

All chemicals and solvents were purchased from Sigma-Aldrich and used as received unless specified. Anhydrous solvents were acquired from a solvent purification system (LC Technology Solutions Inc., SP-1). Silica gel flash column chromatography was performed using an automated CombiFlash® Rf 200 system. Polymer **1** was analyzed by GPC using a Agilent 1100 Series HPLC system equipped with RI, Agilent 1260 Light Scattering and PDA detectors and a

Waters Styragel HR 2 size-exclusion 22 column with 0.1% LiBr/DMF as eluent and flow rate of 1 mL/min at 37 °C. Monodisperse poly(methylmethacrylate) (PMMA) standards were used to determine the molecular weight and PDI of polymer **1**. ^1H NMR and ^{13}C NMR spectra were acquired using a Varian spectrometer working at 600 MHz and 150 MHz respectively. Chemical shifts (δ) are reported in ppm relative to TMS, and coupling constants (*J*) are reported in hertz. High-resolution mass spectra were acquired using an Agilent 6230 ESI-TOFMS in positive ion mode. Irradiation with UV light was done with a Luzchem LZC-ORG photoreactor equipped with 8 UV-A lamps with a power of 1.35 mW/cm^2 and a 10 mW/cm^2, λex = 320-480 nm, OmniCure S2000 Curing System. Particles were formulated using a Qsonica Sonicator 4000 and purifiedby tangential flow filtration Millipore Pellicon XL, 500 kDa. Particles were characterized by DLS, Malvern Instruments Nanosizer, scanning electron microscopy (SEM, Agilent 8500 FE-SEM), and transmission electron microscopy (TEM, Tecnai FEI Spirit). Fluorescence was measured using a Horiba Jobin Yvon FL-1000 fluorimeter and in cells a Molecular Devices SpectraMax M5 plate reader. Fluorescence microscopy images were acquired with a Nikon TS100F.

2. Abbreviations

EDC = 1-ethyl-3-(3-dimethylaminopropyl)carbodiimide, DMAP = 4-dimethylaminopyridine, TFA = trifluoroacetic acid, Et_3N = triethylamine, DMSO = dimethyl sulfoxide, FDA = fluorescein diacetate, DMF = dimethylformamide, TEM = transmission electron microscopy, PDI = polydispersity index, UV = ultraviolet, DLS = dynamic light scattering, GPC = gel permeation chromatography, HPLC = high pressure liquid chromatography, RI = refractive index, PDA = photodiode array.

3. Synthesis of Polymer 1

Compound 3. Compound **6** (1.00 g, 4.7 mmol), compound **2**· dicyclohexylamine (3.25 g, 6.2 mmol) and DMAP (1.15 g, 9.38 mmol) were dissolved in DCM (48 mL). EDC (1.46 g, 9.4 mmol) was added to the solution dropwise and the mixture was allowed to react for 14 h. The reaction mixture was diluted with DCM (50 mL) and extracted 3 times (100 ml) with 1 M HCl. The organic layer was dried over $MgSO_4$ and concentrated. The resulting yellow oil was purified by silica column (3:7 Ethyl Acetate/ Hexane) to yield compound **3** as a yellow solid (1.32 g, 52 %).

HRMS: composition: C_{25} H_{39} N_3 O_{10}; measured mass 564.2527; theoretical mass 564.2528.

^{1}H NMR (600 MHz, $CDCl_3$) δ 7.70 (s, 1H), 7.04 (s, 1H), 5.60 (d, J = 14.9 Hz, 1H), 5.51 (d, J = 14.9 Hz, 1H), 5.21-5.12 (m, 1H), 4.62 (bs, 1H), 4.36-4.28 (m, 1H), 4.00 (s, 3H), 3.94 (s, 3H), 3.15-3.01 (m, 2H), 1.94 – 1.79 (m, 2H), 1.77 – 1.64 (m, 2H), 1.53-1.44 (m, 2H), 1.43 (s, 9H), 1.40 (s, 9H).

^{13}C NMR (151 MHz, $CDCl_3$) δ 172.7, 156.3, 155.76, 153.9, 148.4, 139.8, 127.1, 110.5, 108.4, 80.1, 79.3, 64.0, 56.8, 56.5, 53.8, 40.0, 34.0, 29.8, 28.5, 28.4, 22.6.

Compound 4. Compound 3 (2.071 g, 3.82 mmol) was dissolved in DCM (5 mL) and TFA (5 mL) and stirred for 1 h. The reaction mixture was concentrated. The resulting yellow oil was dissolved in DCM (10 ml) and concentrated 3 more times. The yellow oil was then dissolved in DCM (18.5 mL) and DMF (18.5 mL) and chilled to 0 ^{o}C. Et_3N (3.19 mL, 22.9 mmol) was dripped into the solution slowly. Acryloyl chloride (0.68 mL, 8.41 mmol) was dripped into the reaction mixture over a period of 10 min. The

reaction was quenched after 1 h by addition of 0.05 M HCl (50 mL). The mixture was extracted 3 times with DCM (75 mL). The combined organic was dried over $MgSO_4$ and concentrated. The resulting yellow oil was purified by silica column (3% MeOH in DCM) to yield compound **4** as a yellow solid (0.837 g, 48.5 %).

HRMS: composition: C_{21} H_{27} N_3 O_8; measured mass 472.1692; theoretical mass 472.1690.

^{1}H NMR (600 MHz, DMSO) δ 8.58 (d, *J* = 7.1 Hz, 1H), 8.07 (t, *J* = 5.5 Hz, 1H), 7.71 (s, 1H), 7.19 (s, 1H), 6.32 (dd, *J* = 17.1, 10.2 Hz, 1H), 6.18 (dd, *J* = 17.1, 10.2 Hz, 1H), 6.11 (dd, *J* = 17.1, 2.0 Hz, 1H), 6.05 (dd, *J* = 17.1, 2.2 Hz, 1H), 5.65 (dd, *J* = 10.2, 2.0 Hz, 1H), 5.55 (dd, *J* = 10.2, 2.2 Hz, 1H), 5.48 – 5.41 (m, 2H), 4.42 – 4.34 (m, 1H), 3.93 (s, 3H), 3.87 (s, 3H), 3.16 – 3.06 (m, 2H), 1.83 – 1.75 (m, 1H), 1.74-1.65 (m, 1H), 1.51 – 1.40 (m, 2H), 1.39-1.30 (m, 2H).

^{13}C NMR (151 MHz, DMSO) δ 171.8, 164.9, 164.4, 153.4 147.86, 139.34, 131.82, 130.9, 126.4, 124.7, 110.7, 108.2, 99.5, 63.0, 56.3, 56.1, 52.3, 38.1, 30.3, 28.6, 22.8.

Polymer 1. Compound **4** (150 mg, 0.33 mmol) and Compound **5** (90 mg, 0.33 mmol) were dissolved in DMSO (0.9 mL). Triethylamine (0.28 mL, 2.00 mmol) then 1,3-propanedithiol (72 mg, 0.67 mmol) were added to the solution dropwise. The reaction was stirred at room temperature for 140 h. The reaction mixture was quenched by precipitating with cold ether (40 mL). The polymer was further purified by dissolving in DCM and precipitating into cold ether three (40 mL) times to yield yellow polymer (131 mg, 42 %). M_w of 13900 Da, PDI of 1.71 (characterized by GPC relative to poly(methyl methacrylate) standards).

^{1}H NMR (600 MHz, d_6 DMSO) δ 8.39-8.34 (m 1H), 7.97-7.90 (m, 2H), 7.85 (s, 1H), 7.70 (s, 1H), 7.19 (s, 1H), 5.42 (s, 2H), 4.29 (m, 1H), 3.94 (s, 3H), 3.866 (s, 3H),3.40-3.30 (m, 4H), 3.19-3.13 (m, 4H), 3.05-2.97 (m, 2H), 2.81-2.61 (m, 8H), 2.61-2.51 (m, 8H), 2.47-2.27 (m, 8H), 1.91-1.60 (m, 6H), 1.46-1.29 (m, 4H), 1.28-1.23 (s,6H).

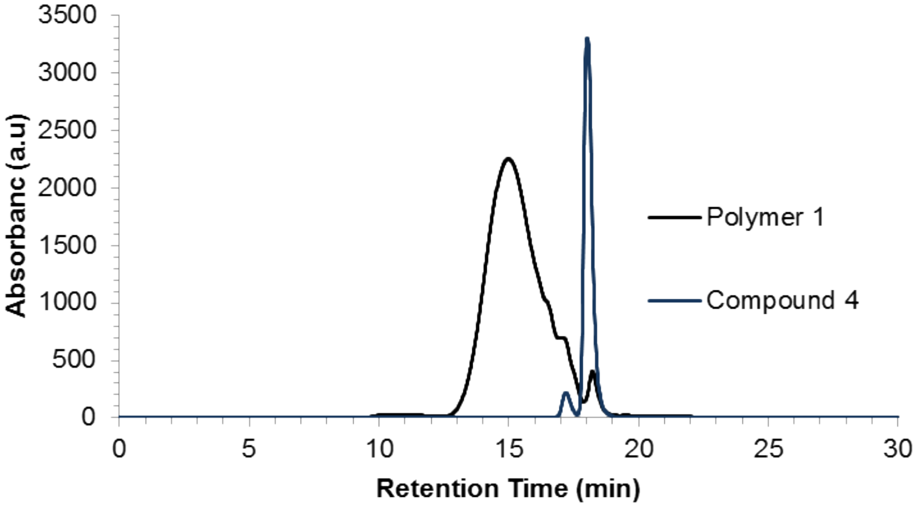

Figure S1: GPC chromatogram of polymer **1** and compound **4**.

4. Degradation of polymer 1

Degradation of polymer 1 followed ^{1}H NMR. A concentrated solution of **polymer 1** (12.5 mg/mL) was prepared in d_6-DMSO. The DMSO stock was divided and an appropriate amount of deuterated sodium phosphate buffer at pH 7.4 (0.1 M) and sodium phosphate solution at pH 5 were added to make 9:1 solutions of d_6-DMSO:PBS. The solution was irradiated for 1 to 20 min in a 1.7 mm Bruker NMR tube in a Luzchem photoreactor. The samples were then incubated at 37 ^{o}C for the specified times. Spectra were taken on a 600 MHz Bruker spectrometer after the prescribed incubation times.

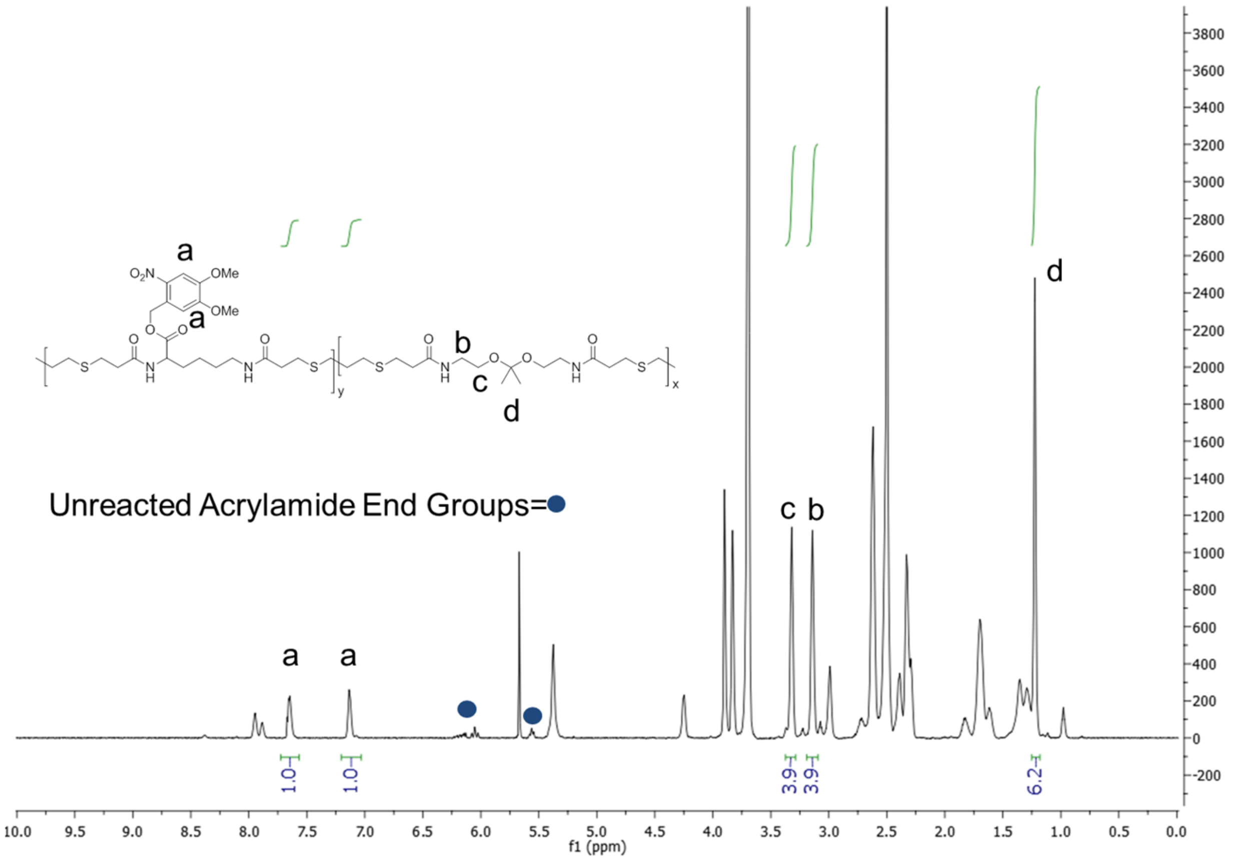

Figure S2: ^{1}H NMR spectra of polymer **1** prior to irradiation in 9:1 solutions of d_6-DMSO:deuterated PBS pH 7.4. Incorporation of monomers **4** and **5** is seen to be roughly 1:1 by comparing the protons of **a** to those of **b** and **c**.

A

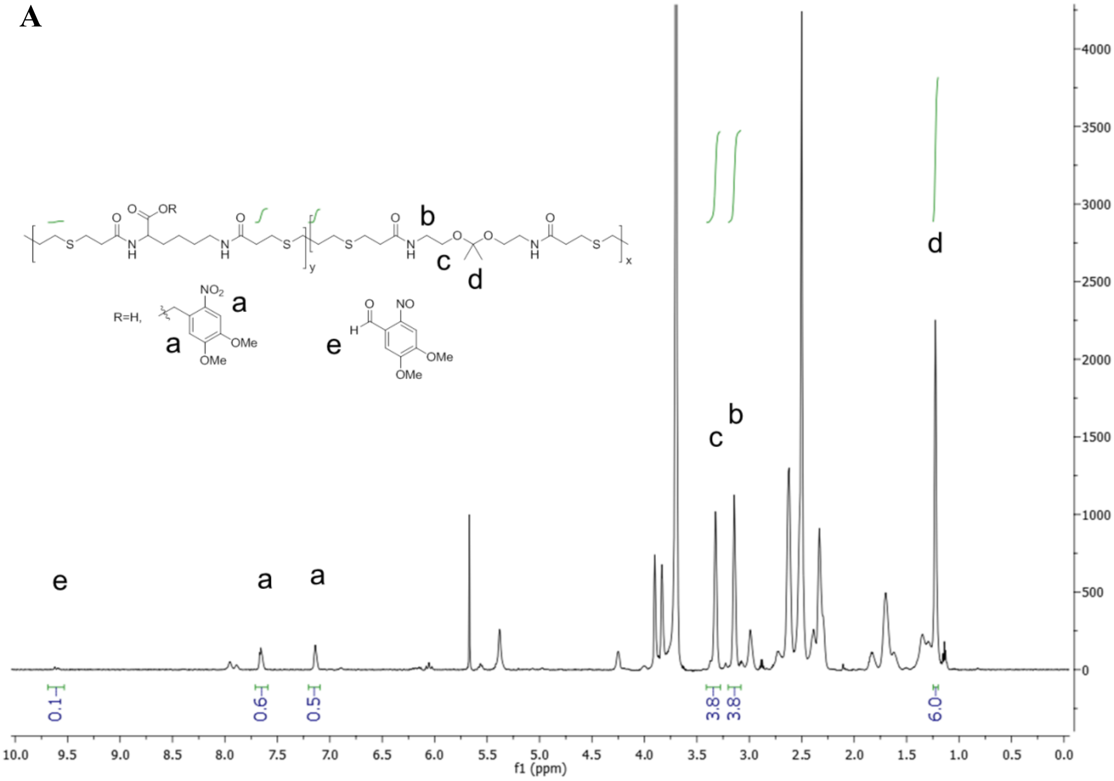

NO2
R=H,
OMe
OMe
NO
OMe
OMe
a
a
e
b
c
d
e
a
a
c
b
d
0.1
0.6
0.5
3.8
3.8
6.0
f1 (ppm)

B

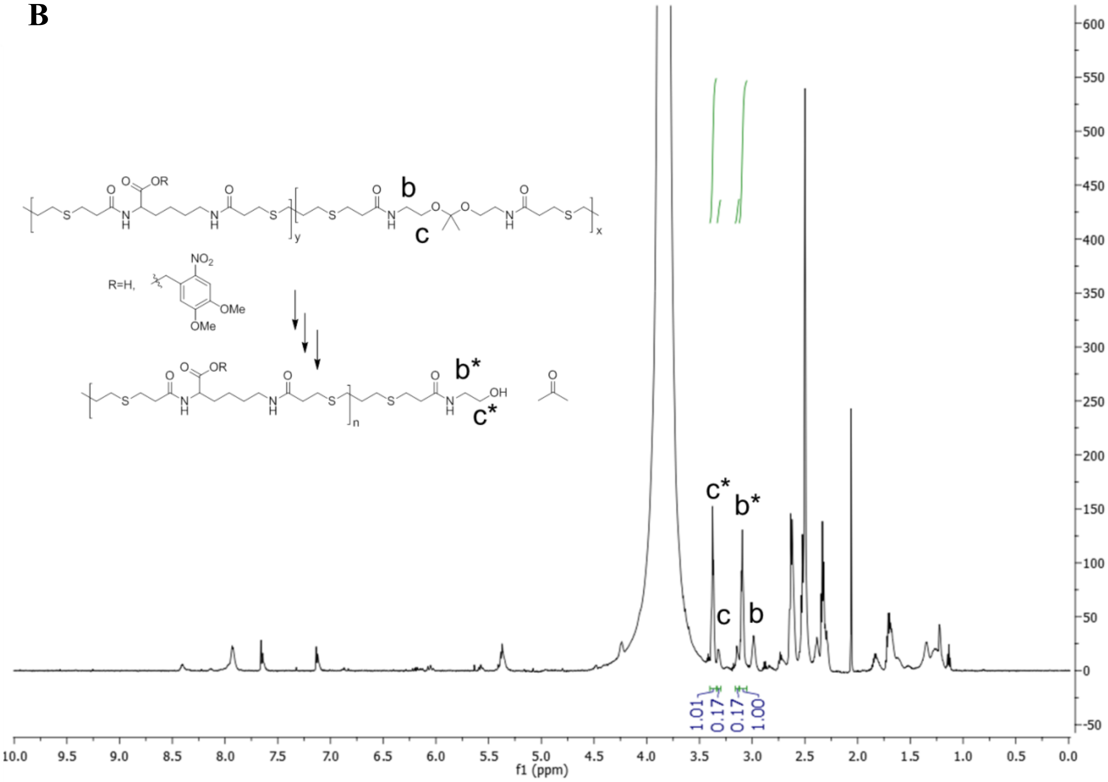

b
c
R=H,
NO2
OMe
OMe
b*
c*
OH
c*
b*
c
b
1.01
0.17
0.17
1.00
f1 (ppm)

C

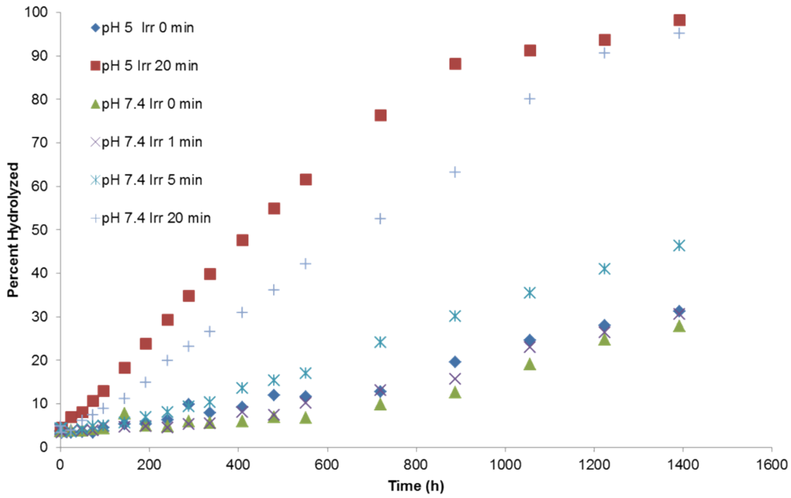

Figure S3: Degradation of polymer 1 followed by ^{1}HNMR. (A-B), ^{1}H NMR spectra of polymer **1** (A) following 20 min irradiation (1.35 mW/cm^2), or (B) following 20 min irradiation and 1224 h incubation in 9:1 solutions of d_6-DMSO:deuterated PBS pH 7.4. (C) Percent hydrolysis of the polymer following the indicated periods of irradiation and incubation. The low quantity of aldehyde observed in (A) is due to its poor solubility in the solvent mixture. To determine the percent of photolyzed protecting group, we compared the signal from unreacted protecting groups **a** to that of backbone protons **c** and **b**.

Degradation of polymer 1 followed GPC. Polymer **1** was dissolved in a mixture of acetonitrile and PBS 90:10. Two solutions were prepared with pH 7.4 (0.1 M) PBS. One of the samples with PBS pH 7.4 was irradiated in a Luzchem photoreactor for 20 min. The samples were incubated at 37 ^{o}C for the 0, 24, 115, and 336 h. At the given times the samples were concentrated at 30 ^{o}C, dissolved in DMF with 0.01% LiBr, and analyzed by gel permeation chromatography monitoring at 320 nm.

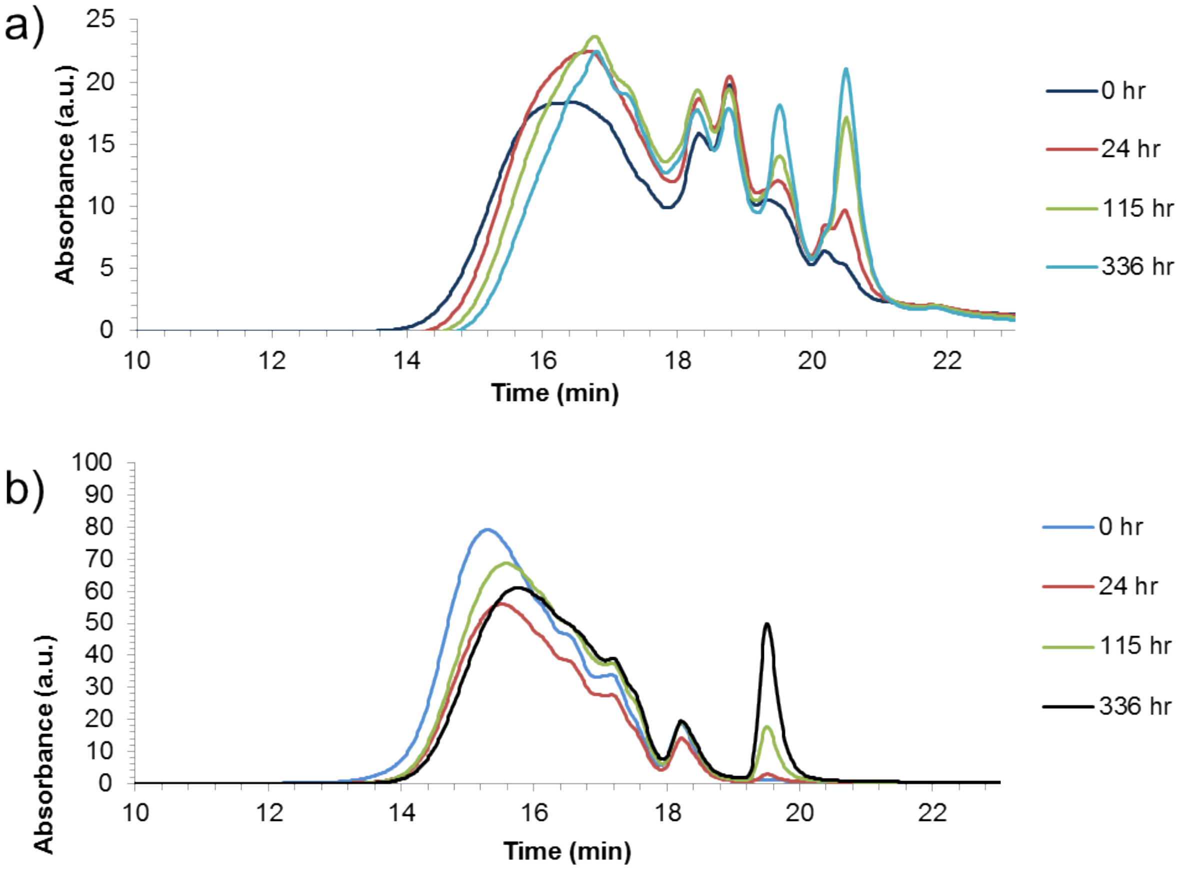

Figure S4: GPC chromatograms of an a) irradiated and b) control (not irradiated) sample of polymer **1** after 0, 24, 115, and 336 h of incubation at 37 ^{o}C.

5. Particle formulation and characterization

Particle formulation. Polymer 1 (10 mg) was dissolved in DCM (270 µL), and combined with fluorescein diacetate (FDA, 2mg) in DMSO (30 µL), or Nile Red (NR, 1 mg) in DCM (30 µL). The resulting solution was added to sterile-filtered polyvinyl alcohol (PVA, 1% w/v) in 10 mM Tris pH 8.0 buffer (6 mL), and probe sonicated for 4 min at 9-10W (Qsonica Sonicator 4000). DCM was then removed by stirring at 600 RPM under house vacuum for 3 h. Remaining PVA was removed by tangential flow filtration (Millipore Pellicon XL, 500 kDa) using 10 mM Tris pH 8.0 buffer (250 mL) at 45 RPM. The retentiate was freeze-dried with 100 mg trehalose as cryoprotectant. The size and distribution of particles were determined by dynamic light scattering (DLS, Malvern Instruments

Nanosizer) and transmission electron microscopy (TEM, Tecnai FEI Spirit). Loading and encapsulation efficiency was assessed by dissolving lyophilized powder in DCM, extracting FDA into NaOH (0.1 N) and measuring fluorescence of the aqueous phase against a calibration curve in 0.1 N NaOH (Horiba Jobin Yvon FL-1000).

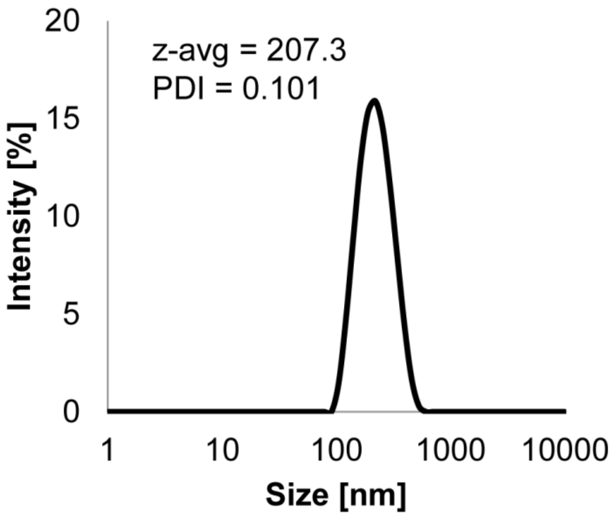

Figure S5: DLS size distribution of formulated particles of Polymer **1** containing Nile Red.

Particle degradation. Freeze-dried particle powder was re-suspended in Tris pH 7.4 buffer and irradiated for 5 min (35 mW/cm^2, λex = 320-480 nm, Lumen Dynamics Omnicure S2000 Curing System). Particle degradation was assessed by DLS, SEM, and TEM at different timepoints compared to the non-irradiated sample.

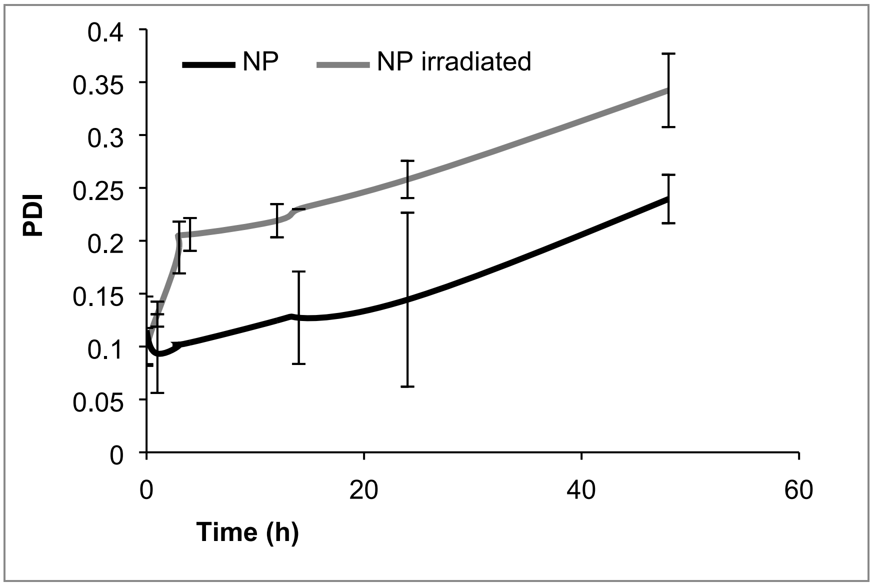

Figure S6: PDI of nanoparticles after irradiation 5 min (35 mW/cm^2, λ = 320-480 nm) by DLS. The initial rapid increase in PDI in irradiated samples is related to swelling and degradation caused by irradiation. Later changes are the result of slower subsequent hydrolysis and aggregation.The PDI of non-irradiated particles also slowly increases over time due to slow hydrolysis and subsequent aggregation.

Nile Red quenching. Freeze-dried Nile Red-containing particles were re-suspended in 1x PBS pH 7.4 buffer (1 mg/mL). Quenching of Nile Red fluorescence upon degradation and solubility change was followed by the decrease in the 620 nm fluorescence peak (Horiba Jobin Yvon FL-1000). Suspensions were irradiated for 1, 3, or 5s intervals (1.5 mW/cm^2, 300-400 nm, λ_{max} = 365 nm), incubating 10 min in between each consecutive irradiation.

6. *In vitro* release from particles

Release in 264.7 Raw cells. Raw 264.7 mouse macrophage cells were seeded at 20000 cells/well in DMEM (Corning) supplemented with 10% Fetal Bovine Serum (FBS, HyClone) and 1% penicillin-streptomycin (Invitrogen) on a 96-well tissue culture-treated plate (Corning) overnight prior to the experiment. Lyophilized FDA-containing particles (10 mg) were resuspended in clear DMEM/FBS (1 mL, no phenol red, Corning). The cells were double washed with warm PBS (100 µL twice) and the particle suspensions in media were added (100 µL). Free FDA (0.1 mg/mL) and empty particles were

used as controls. After 3h incubation at 37 °C, in 5% CO_2 atmosphere, the cells were again double washed with warm PBS (100 µL twice) and clear media (100 µL) was added to each well. Half of the plate was then irradiated for 5 min (10 mW/cm^2, λex = 320-480 nm) using the Omnicure. Following a 30 min incubation at 37 °C, in 5% CO_2, the release of FDA was measured by fluorescence measurement (λex = 495 nm, λem = 514 nm) using a plate reader (Molecular Devices SpectraMax M5). To confirm release, fluorescence microscopy images of cells were acquired (Nikon TS100F).

7. Cytotoxicity assays

Polymer Cytotoxicity. Polymer 1 (5 mg) was dissolved in sterile DMSO (10 µL), and the solution was subsequently added to clear DMEM (990 µL). The resulting suspension was sonicated until uniform and then further diluted to appropriate concentrations in DMEM/FBS media. Lyophilized particles containing FDA (5 mg) were resuspended in sterile media (1 mL) and half of the volume was irradiated for 5 min with UV light (10 mW/cm^2, λex = 320-480 nm, OmniCure S2000 Curing System). The solutions were then diluted to appropriate concentrations in cell culture media. 24 hours prior to incubation, Raw 264.7 cells were seeded on a tissue culture treated 96-well plate (Corning) at a density of 20000 cells/well in DMEM media. The cells were washed twice with 100 µL PBS at 37 °C, and then incubated with the polymer/particle suspensions in triplicates for 24 h at 37 °C, in 5% CO_2. Following incubation with particles, cells were again washed twice with 100 µL PBS. Following the MTT assay kit instructions, the cells were then incubated at 37°C, in 5% CO_2 for 3 h in 100 µL DMEM containing 0.5 mg/mL 3-[4,5-dimethylthiazol-2-yl]-2,5-diphenyl tetrazolium bromide (MTT agent, Sigma-Aldrich, USA). Triton-X (1% w/v, Sigma-Aldrich) was used as a positive apoptosis control. After the incubation, 100 µL of MTT solution (Sigma-Aldrich) was added to each well and the solution was thoroughly triturated to fully solubilize formazan crystals. To quantify mitochondrial activity, absorbance at 570 nm normalized to background absorbance at 690 nm was measured using a plate reader (Molecular Devices SpectraMax M5).

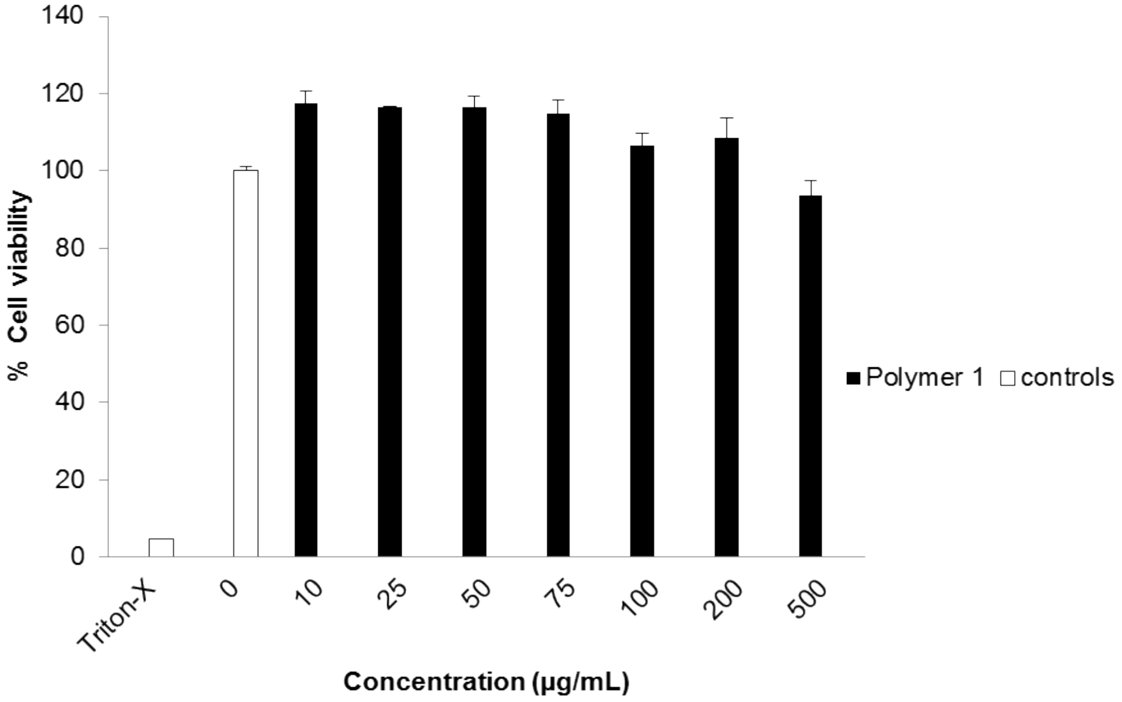

Figure S7: MTT assay of polymer **1**.

Particle Cytotoxicity.

Lyophilized empty **polymer 1** nanoparticles were re-suspended in sterile DMEM/FBS media (1 mL) and diluted to appropriate concentrations in cell culture media. 24 hours prior to incubation, Raw 264.7 cells were seeded on a tissue culture treated 96-well plate (Corning) at a density of 20000 cells/well in DMEM media. The cells were washed twice with 100 µL PBS at 37 °C, and then incubated with the particle suspensions in triplicates for 3 h at 37 °C, in 5% CO_2. Following incubation with particles, half of the plate was irradiated for 5 min with UV light (10 mW/cm^2, λex = 320-480 nm, OmniCure S2000 Curing System). Cells were then incubated for an additional 24h at 37 °C, in 5% CO_2. Subsequently, cells were washed twice with 100 µL PBS. Following the MTT assay kit instructions, the cells were then incubated at 37°C, in 5% CO_2 for 3 h in 100 µL DMEM containing 0.5 mg/mL 3-[4,5-dimethylthiazol-2-yl]-2,5-diphenyl tetrazolium bromide (MTT agent, Sigma-Aldrich, USA). Triton-X (1% w/v, Sigma-Aldrich)

was used as a positive apoptosis control. After the incubation, 100 µL of MTT solution (Sigma-Aldrich) was added to each well and the solution was thoroughly triturated to fully solubilize formazan crystals. To quantify mitochondrial activity, absorbance at 570 nm normalized to background absorbance at 690 nm was measured using a plate reader (Molecular Devices SpectraMax M5).

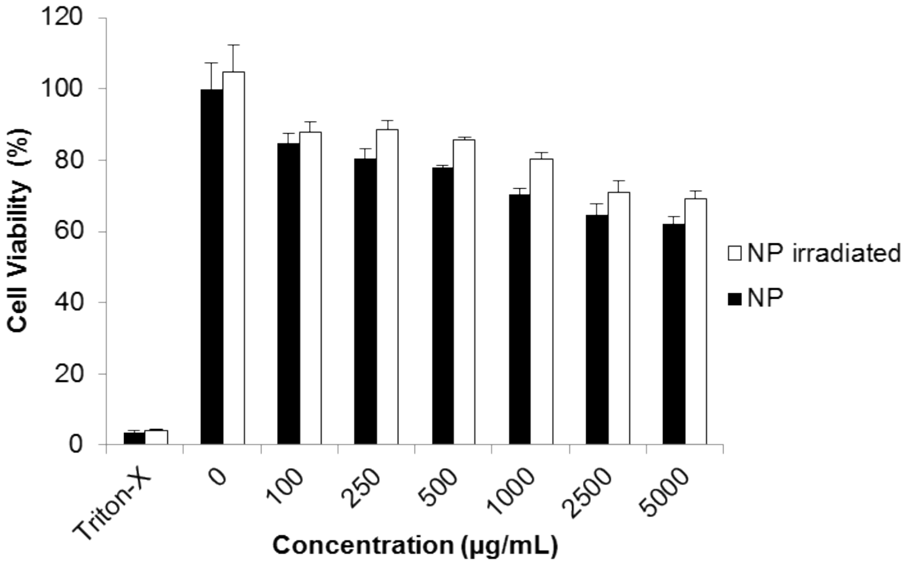

Figure S8: MTT assay of cells incubated with particles composed of polymer 1 and irradiated for 5 min with UV light at 10 mW/cm^2.

8. Synthesis of control polymer 9

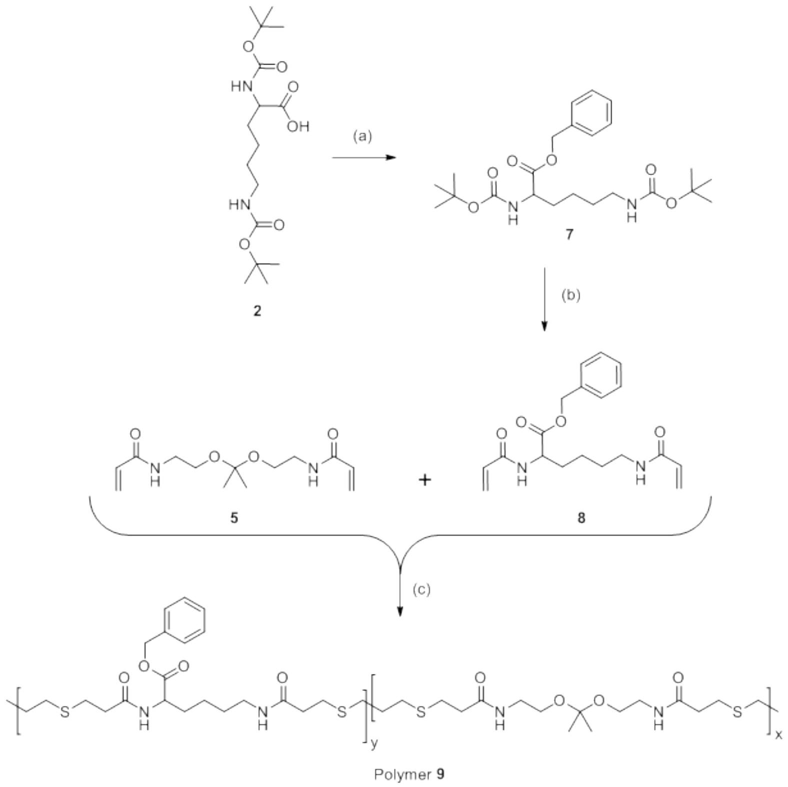

Figure S9: Synthesis of polymer **9**. a) benzyl bromide, cesium carbonate, DMF, 99%; b) i) TFA, DCM ii) acryloyl chloride, Et_3N, DCM, 0 ^oC, 61%; c) **5**, 1,3-propanedithiol, Et_3N, DMSO, 39%.

Compound 7. Compound **2**· dicyclohexylamine (2.00 g, 3.79 mmol) and cesium carbonate (2.07 g, 6.35 mmol) were suspended in DMF (30 mL). Benzyl bromide (0.75 mL, 6.35 mmol) was added to the solution dropwise and the mixture was allowed to react for 18 h. The reaction mixture was diluted with DI H_2O (80 mL) and extracted 3 times with DCM (50 mL). The organic layer was dried over $MgSO_4$ and concentrated. The resulting colorless oil was purified by silica column (1:3 Ethyl Acetate/ Hexane) to yield compound **7** as a colorless oil (1.65 g, 99 %).

HRMS: composition: C_{25} H_{36} N_2 O_6Na; measured mass 459.2464; theoretical mass 459.2466.

^{1}H NMR (600 MHz, $CDCl_3$) δ 7.46-7.31 (m, 5H), 5.20 (d, J = 12.6 Hz, 1H), 5.14-5.02 (m, 2H), 4.52 (bs, 1H), 4.32 (bs, 1H), 3.16-2.99 (m, 2H), 1.88-1.76 (m, 1H), 1.76-1.60 (s, 2H), 1.43 (s, 18H), 1.37-1.24 (m, 3H).

^{13}C NMR (151 MHz, $CDCl_3$) δ 172.6, 156.0, 155.4, 135.4, 128.7, 128.6, 128.5, 80.0, 79.3, 67.1, 53.5, 40.2, 32.5, 29.7, 28.6, 28.5.

Compound 8. Compound 7 (1.65 g, 3.78 mmol) was dissolved in DCM (18 mL) and TFA (18 mL) and stirred for 1.2 h. The reaction mixture was concentrated. The resulting colorless oil was dissolved in DCM (40 mL) and concentrated 3 more times. The oil was then dissolved in DCM (37 mL) and Et_3N (4.32 mL, 30.98 mmol) was dripped into the solution slowly and the solution was chilled to 0 ^{o}C. Acryloyl chloride (0.69 mL, 8.52 mmol) was dripped into the reaction mixture over a period of 10 min. The reaction was quenched after 1 h by addition of 0.05 M HCl (50 mL). The mixture was extracted 3 times with DCM (40 mL). The combined organic was dried over $MgSO_4$ and concentrated. The resulting yellow oil was purified by silica column (3:2 Ethyl Acetate/ Hexane) to yield compound **8** as a white solid (0.818 g, 61.3 %).

HRMS: composition: C_{19} H_{25} N_2 O_4; measured mass 345.1807; theoretical mass 345.1809.

^{1}H NMR (600 MHz, $CDCl_3$) δ 7.40-7.32 (m, 5H), 6.41 (d, J = 7.8 Hz, 1H), 6.32 (d, J = 16.8 Hz, 1H), 6.27 (d, J = 16.8 Hz, 1H), 6.18 (dd, J = 16.8, 10.2 Hz, 1H), 6.08 (dd, J = 16.8, 10.2 Hz, 1H), 5.84 (bs, 1H), 5.69 (d, J = 10.2 Hz, 1H), 5.62 (d, J = 10.2 Hz, 1H), 5.21 (d, J = 12.6 Hz, 1H), 5.16 (d, J = 12.6, 1H), 4.74 – 4.67 (m, 1H), 3.36 – 3.23 (m, 2H), 1.94 – 1.86 (m, 1H), 1.81 – 1.72 (m, 1H), 1.60 – 1.49 (m, 2H), 1.44 – 1.25 (m, 2H).

^{13}C NMR (151 MHz, DMSO) δ 172.4, 166.1, 165.7, 135.4, 131.0, 130.5, 128.8, 128.6, 128.4, 127.3, 126.4, 67.4, 52.0, 38.8, 32.0, 28.7, 22.3.

Polymer 9. Compound **8** (76 mg, 0.22 mmol) and Compound **5** (59 mg, 0.22 mmol) were dissolved in DMSO (0.9 mL). Triethylamine (0.18 mL, 1.34 mmol) then 1,3-propanedithiol (47 mg, 0.44 mmol) were added to the solution dropwise. The reaction was stirred at room temperature for 336 h. The reaction mixture was diluted with DCM and quenched by precipitation into cold ether (40 mL). The polymer was further purified by dissolving in DCM and precipitating into cold ether (40 mL) three times to yield colorless polymer **9** (69 mg, 38 %). M_w of 3500 Da, PDI of 1.62 (characterized by GPC relative to poly(methyl methacrylate) standards).

^1H NMR (600 MHz, d_6 DMSO) δ 8.19-8.13 (m 1H), 8.08-8.03 (m, 1H), 7.96-7.90 (s, 1H), 7.86-7.82 (s, 1H), 7.41-7.30 (m, 5H), 5.12 (s, 2H), 4.29-4.22 (m, 1H), 3.43-3.36 (m, 3H), 3.28-3.22 (m, 1H), 3.20-3.12 (m, 2H), 3.12-3.05 (m, 1H), 3.03-2.96 (m, 1H), 2.82-2.78 (m, 4H), 2.70-2.62 (m, 6H), 2.60-2.52 (m, 8H), 2.45-2.28 (m, 6H), 2.04-1.95 (m, 1H), 1.91-1.83 (m, 2H), 1.78-1.66 (m, 2H), 1.64-1.55 (m, 1H), 1.44-1.33 (m, 2H), 1.34-1.21 (m, 8H) 1.05-0.94 (m, 2H).

9. Degradation of control polymer 9

Deprotection of polymer 9 via hydrogenation. Polymer **9** (15 mg) was dissolved in THF (4 mL) under argon. Pd/C 10% (10 mg) was added and the reaction was put under an H_2 atmosphere. The reaction was allowed to proceed for 15 h then was flushed with argon, filtered through celite, then concentrated. The resulting oil was used directly in ^1H NMR experiments. The hydrogenation appeared to remove roughly 50% of the protecting group (Figure S8). This is likely due to poisoning of the palladium catalyst with residual thiols.

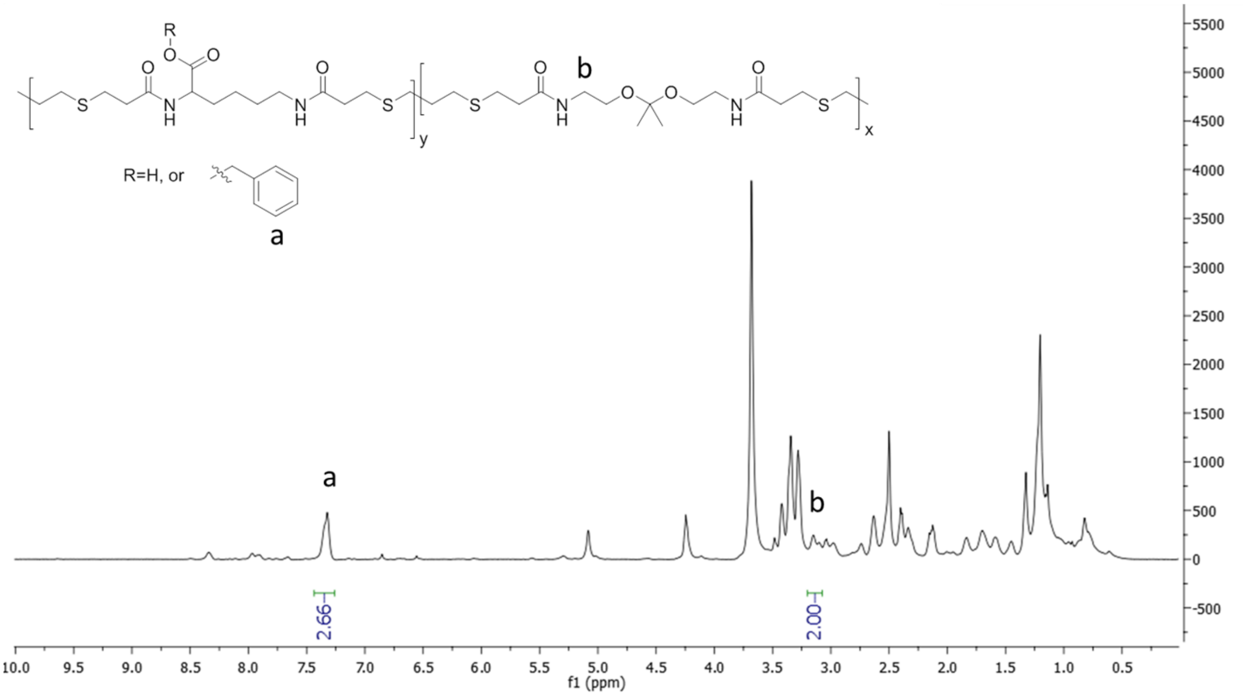

Figure S10: Hydrogenated polymer **9**.

Degradation of polymer 9 followed ^{1}H NMR. A concentrated solution of polymer **9** (12.5 mg/ mL) was prepared in d_6-DMSO. An appropriate amount of deuterated sodium phosphate buffer at pH 7.4 (0.1 M) was added to make 9:1 solutions of d_6-DMSO:PBS and was separated to two samples. One sample was irradiated for 20 min in a 1.7 mm Bruker NMR tube in a Luzchem photoreactor. One sample of polymer **9** was not irradiated. Hydrogenated polymer **9** was prepared in d_6-DMSO (22 mg/ mL) and an appropriate amount of deuterated sodium phosphate buffer at pH 7.4 (0.1 M) was added to make a 9:1 solutions of d_6-DMSO:PBS solution. The samples were then incubated at 37 ^{o}C for the specified times. Spectra were taken on a 600 MHz Bruker spectrometer after the prescribed incubation times.

a)

Control Polymer	Initial Rate (10^{-6} mol L^{-1} h^{-1})
Not Irradiated	4.7
Irradiated (20 min)	5.0
Hydrogenated	18.7

b)

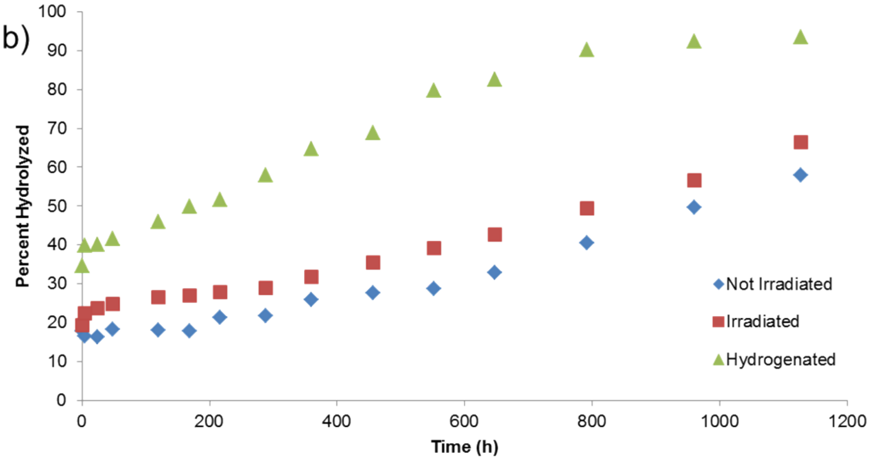

Figure S11: (a) The initial rate of hydrolysis for the ketal calculated based on protons vicinal to the amide (protons b Figure S8) in 9:1 solutions of d_6-DMSO:deuterated PBS pH 7.4. (b) Percent hydrolysis of the polymer following the indicated periods of incubation for polymer **9** (blue diamonds), polymer **9** irradiated for 20 min (red squares), and hydrogenated polymer **9** (green triangles).

10. NMR spectra

Compound 3

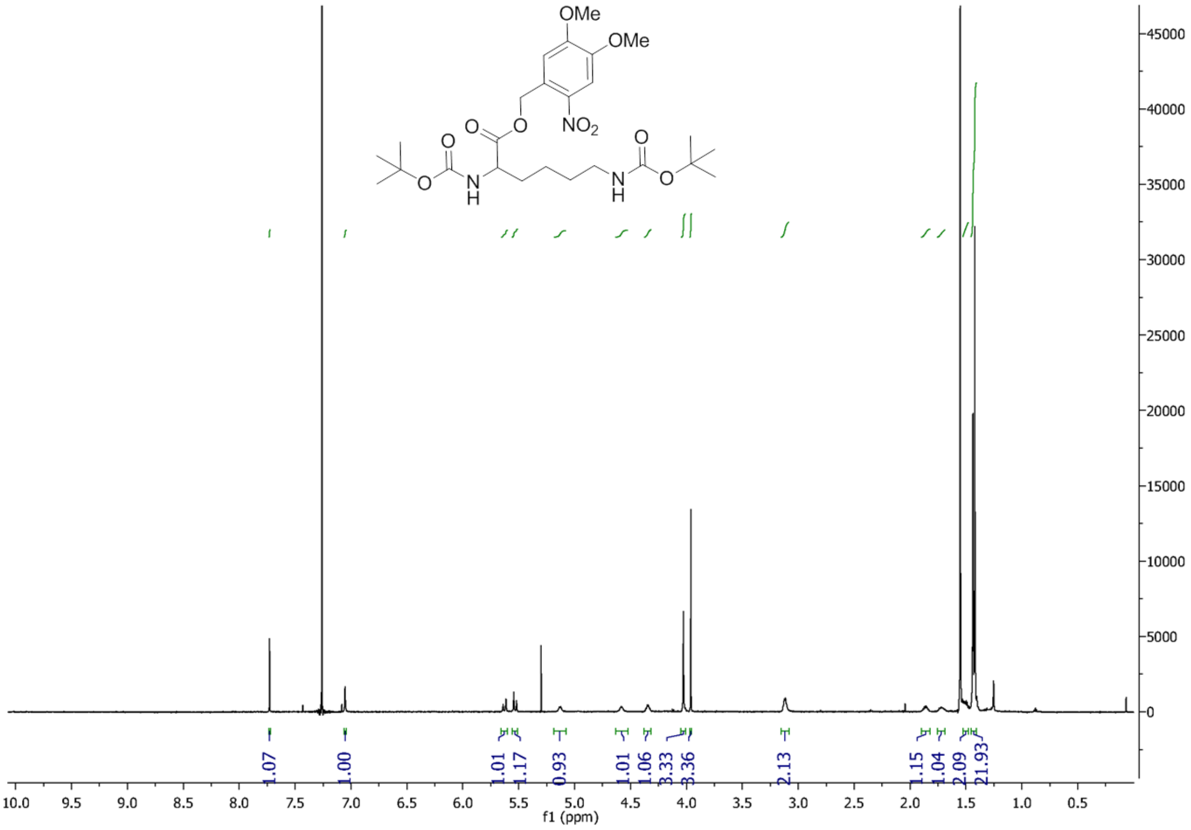

Compound 4

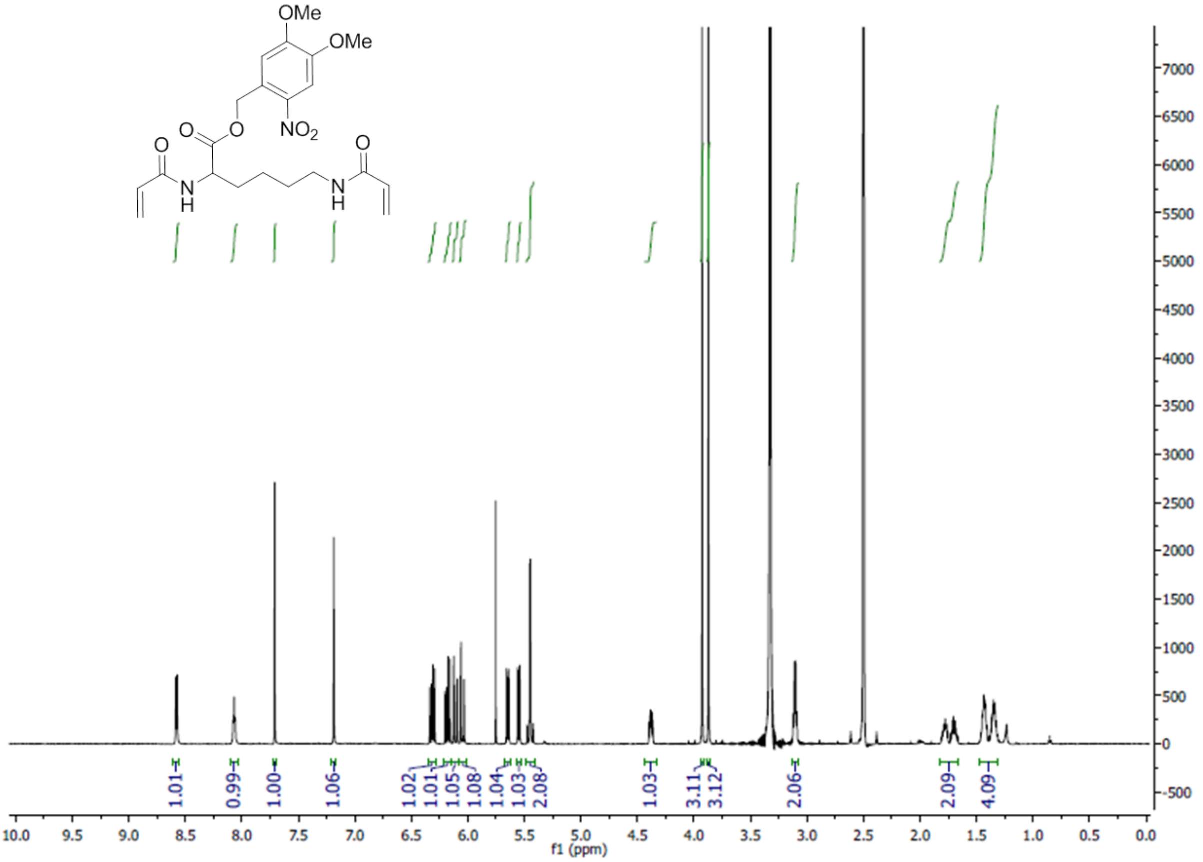

OMe
OMe
NO2
O
O
O
N
H
N
H
1.01
0.99
1.00
1.06
1.02
1.01
1.05
1.08
1.04
1.03
2.08
1.03
3.11
3.12
2.06
2.09
4.09
f1 (ppm)

11. Additional TEM images

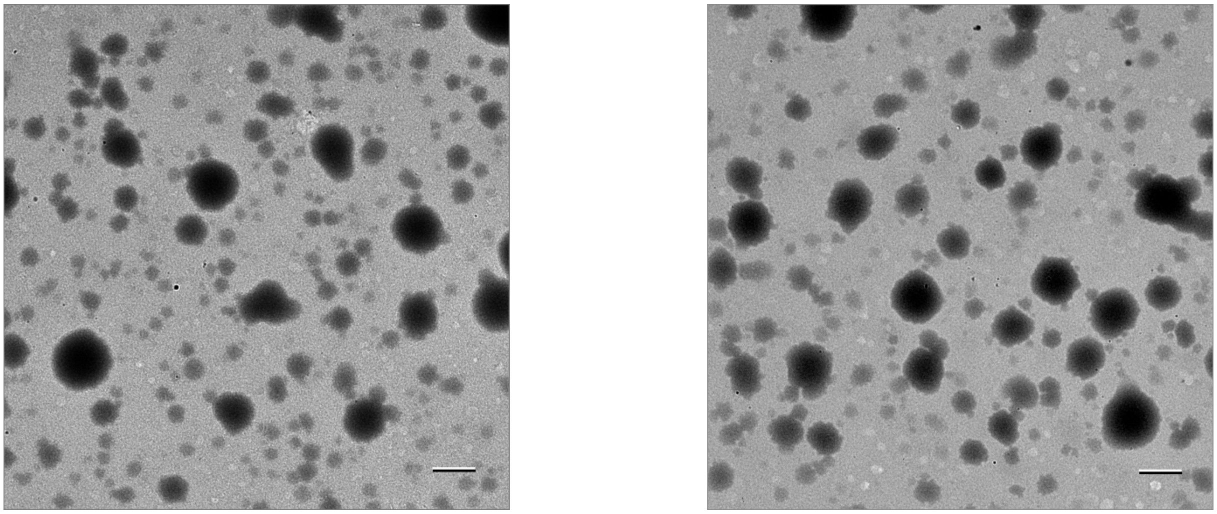

Figure S12: Representative TEM micrographs of particles incubated in the dark at 37 °C for 4 h (scale bars = 500 nm)

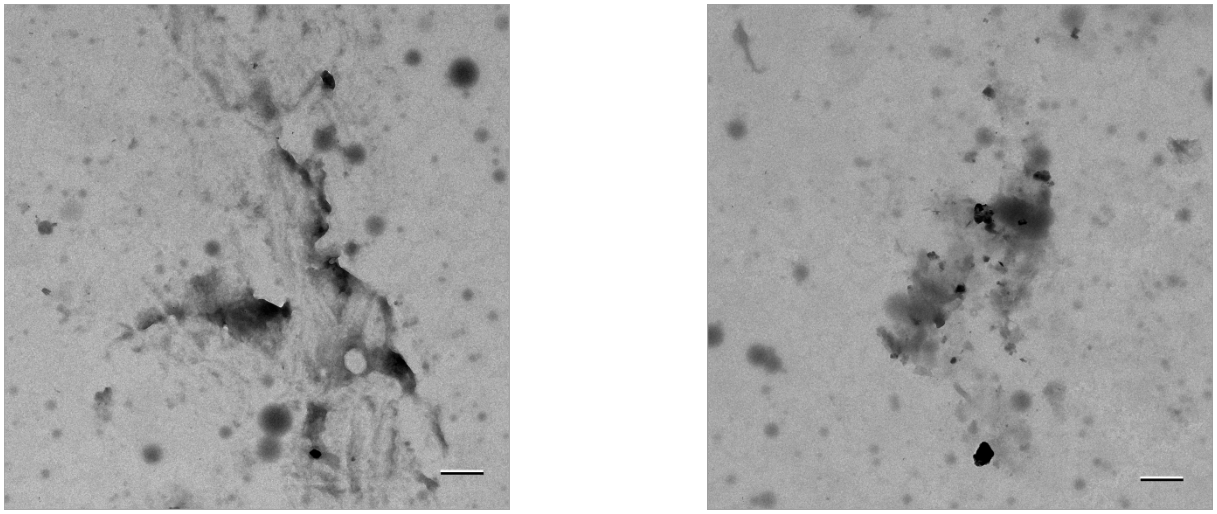

Figure S 13: Representative TEM micrographs of particles post-irradiation for 5 min (35 mW/cm^2, λ = 320-480 nm) and incubation at 37 °C for 4 h. (scale bars = 500 nm)

12. SEM images

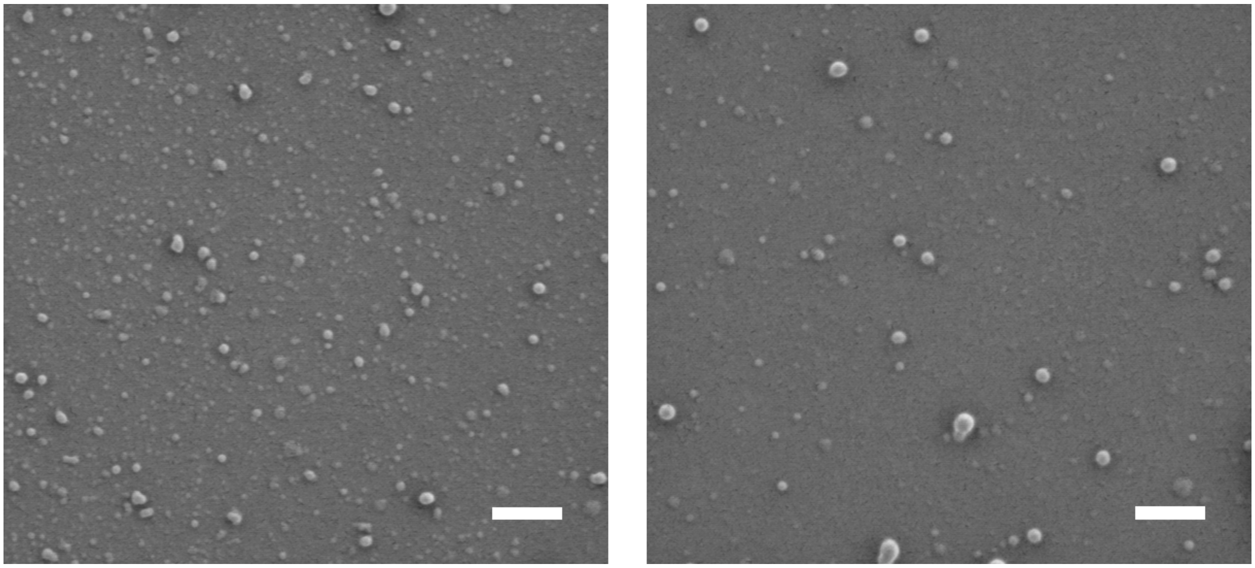

Figure S14: Representative SEM images of empty particles composed of polymer **1** scale Bar = 2 μm.
